# MiR‐145 affected the circular RNA expression in prostate cancer LNCaP cells

**DOI:** 10.1002/jcb.27181

**Published:** 2018-08-22

**Authors:** Jin‐Hua He, Ze‐Ping Han, Jia‐Bin Zhou, Wei‐Ming Chen, Yu‐Bing Lv, Men Ling He, Yu‐Guang Li

**Affiliations:** ^1^ Department of Laboratory Medicine Central Hospital of Panyu District, Guangzhou Guangdong China

**Keywords:** circular RNA (circRNA), microarray, miR‐145, prostate cancer (Pca)

## Abstract

Recent evidence has demonstrated that circular RNAs (circRNAs) played crucial roles in fine‐tuning the levels of gene expression by sequestering the corresponding microRNA (miRNAs). Their interaction with disease‐associated miRNAs indicates that circRNAs are important for the development of disease, and miR‐145 has been previously shown to have antitumor effect in prostate cancer. In the current study, we successfully established the miR‐145‐overexpressed prostate cancer LNCaP cells (LNCaP‐miR‐145) using lentiviral vectors. LNCaP cells expressing the empty vector (LNCaP‐NC) were used as the negative control. The circRNA expression in LNCaP‐miR‐145 cells was detected by microarray analysis, and the miRNA targets of circRNAs were predicted using the bioinformatics software TargetScan and miRanda. Quantitative reverse transcription polymerase chain reaction was used to detect the expression levels of circRNAs in the prostate cancer tissue, nonmalignant tissue, LNCaP‐miR‐145 cells, and LNCaP‐NC cells. The interaction of miRNA and circRNA was further confirmed by the dual‐luciferase reporter assay. A total of 267 and 149 circRNAs were significantly up‐ and downregulated in LNCaP‐miR‐145 cells, respectively. hsa_circRNA_101981, hsa_circRNA_101996 and hsa_circRNA_09142 were the 3 circRNAs that interacted with hsa‐miR‐145‐5p; hsa_circRNA_008068 and hsa_circRNA_406557 were the 2 circRNAs that interacted with hsa‐miR‐145‐3p. Most of the circRNAs corresponded to the protein‐coding exons. The expression levels of hsa_circRNA_101981, hsa_circRNA_00806, and hsa_circRNA_406557 were upregulated in the LNCaP‐miR‐145 cells, but downregulated in the prostate cancer tissue. In contrast, the expression levels of hsa_circRNA_101996 and hsa_circRNA_091420 were downregulated in the LNCaP‐miR‐145 cells, but upregulated in the prostate cancer tissue. Moreover, miR‐145‐5P might regulate the expression of hsa_circRNA_101981, hsa_circRNA_101996, and hsa_circRNA_09142, while miR‐145‐3P might regulate the expression of hsa_circRNA_008068 and hsa_circRNA_406557. Overexpression of miR‐145 promoted the expression of hsa_circRNA_101981, hsa_circRNA_008068, and hsa_circRNA_406557 but suppressed the expressions of hsa_circRNA_101996 and hsa_circRNA_091420 in LNCaP cells. The results from the current study should give us a clue to clarify the tumor suppressive effect of miR‐145.

**Abbreviations**
circRNA: circular RNAs
miR or miRNA: microRNA
Pca: prostate cancer
RT‐PCR: reverse transcription polymerase chain reaction

## 1 INTRODUCTION

Prostate cancer (Pca) has become the most common cancer in men in the United States, next only to lung cancer, and has high mortality. It ranks the third leading cause of death in the European Union, only next only to lung cancer and colorectal cancer.^1^ In China, with the aging of the social population, the incidence of Pca is increasing every year. Early diagnosis and treatment are beneficial to the prognosis of Pca.^2^ Therefore, the identification of the predictive biomarkers and targetable molecules for early detection and therapy of Pca are currently the research hotspots in the relevant field.

A previous study has shown that miR‐145 not only could inhibit the proliferation and invasion of Pca LNCaP cells, but was also involved in the regulation of lncRNAPCGEM1.^3^ It has also been shown to play an inhibitory role in neuroblastoma, osteosarcoma, uterine cancer, and Pca,^4^ but the molecular mechanisms involved remain to be further explored.

Unlike the linear RNA, circular RNA (circRNA) is a type of RNA that forms a covalently closed continuous loop.^5^ It is largely expressed in eukaryotic transcriptome and conserved among species with stable expression and tissue/time specificity. It is resistant to exonuclease‐mediated degradation, and is presumably more stable than most linear RNAs in cells. Therefore, it has the potential to be used as a tumor biomarker.^6^, ^7^ A recent study has demonstrated that circRNA could function as a microRNA (miRNA) sponge and participate in the regulation of gene transcription.^8^ A study of colorectal cancer revealed that circRNA001596 functioned as a miRNA sponge and a positive regulator in tumor proliferation and invasion by upregulating the gene expressions of E2F5, BAG4, and FMNL2.^9^ Another study showed that the expression of cir‐ITCH was downregulated in esophageal squamous cell carcinoma tissues, which could further upregulate the ITCH gene expression by binding to miR‐7, miR‐17, and miR‐124. By inhibiting the Wnt signaling pathway, cir‐ITCH functioned as a tumor suppressor in esophageal squamous cell carcinoma.^10^

In the current study, we aim to clarify the role of miR‐145 in the regulation of circRNA expression in cells overexpressing miR‐145, as well as to identify its target circRNA. By conducting this study, we hope to broaden our knowledge of the pathogenesis of Pca and eventually improve its treatment strategy.

## 2 METHODS

### 2.1 Tissue specimens

Clinical tissue specimens from patients with PCa or nonmalignant prostate tissue were obtained from the archives of the Central Hospital of Panyu District with informed consent and with the approval of the institutional Ethics Committee (Guangdong, China). No local or systemic treatments were administered to these patients preoperatively. All of the tissue samples were immediately snap‐frozen in liquid nitrogen after surgical excision and stored at −80°C until total RNA was extracted for further experimentation.

### 2.2 Cell culture

Noncancerous RWPE‐1 and cancerous LNCaP cells were purchased from the Shanghai Institute of Cell Biology (Shanghai, China). The cell lines were cultured in Dulbecco modified Eagle medium (Gibco BRL, Grand Island, NY) containing 10% fetal bovine serum (HyClone; Invitrogen, Camarillo, CA), 100 U/mL of penicillin (Invitrogen), and 100 μg/mL of streptomycin (Invitrogen). Cells were maintained in a humidified incubator at 37°C in the presence of 5% CO_2_. All cell lines were passaged for less than 6 months before used in this study.^3^

### 2.3 Construction of LNCaP cells overexpressing miR‐145

Human complementary DNA was used as the template. The upstream and downstream primers F/R (Hsa‐mir‐145‐5pEcoRIF: 5′ccggaattcCGCCAGAGGGTTTCCGGTACTTTTC 3′; Hsa‐mir‐145‐5pBamHIR: 5′cgcggatccCATCCAGCCTCACAGGGATGTTA 3′) were reacted with the corresponding cleavage site. The purified polymerase chain reaction (PCR) product and the lentiviral vector (pLVX‐mCMV‐ZsGreen‐puro) were then subjected for double digestion with respective enzymes. The enzyme‐digested products were ligated using T4 DNA ligase at 20°C overnight. The ligated products were then used to transfect 293T cells. Six hours after the transfection, the medium was replaced. Forty‐eight hours after the transfection, the medium containing the miR‐145‐expressing lentiviral vectors was then collected and further used for infecting the LNCaP cells in the presence of polybrene. Empty vector infected LNCaP cells were used as negative controls. Forty‐eight hours after the infection, puromycin was added for the selection of the successfully transfected LNCaP cells.

### 2.4 Labeling and hybridization

Ample labeling and array hybridization were performed according to the manufacturer's protocol (Arraystar Inc., Rockville, MD). Briefly, total RNAs were digested with Rnase R (Epicentre, Madison, WI) to remove linear RNAs and enrich circRNAs. Then, the enriched circRNAs were amplified and transcribed into fluorescent complementary RNA utilizing a random priming method (Arraystar Super RNA Labeling Kit; Arraystar Inc., Rockville, MD). The labeled complementary RNAs were purified by the RNeasy Mini Kit (Qiagen, Hilden, Germany). The concentration and specific activity of the labeled cRNAs (pmol Cy3/μg cRNA) were measured by NanoDrop ND‐1000. One microgram of each labeled cRNA was fragmented by adding 5 μL 10 × Blocking Agent and 1 μL of 25 × Fragmentation Buffer, then heated the mixture at 60°C for 30 minutes, finally 25 μL 2 × Hybridization buffer was added to dilute the labeled cRNA. 50 microliter of hybridization solution was dispensed into the gasket slide and assembled to the circRNA expression microarray slide. The slides were incubated for 17 hours at 65°C in an Agilent Hybridization Oven. The hybridized arrays were washed, fixed, and scanned using the Agilent Scanner G2505C. All of the experiments were performed by Shanghai Kang Cheng biology Co., Ltd.

### 2.5 Luciferase reporter assay

The circRNAs were built into PSI‐CHECK2 vector, then circRNA and miRNA were cotransfected into 293T. Luciferase activities were measured at 48‐hours after transfection using the Dual‐Luciferase Reporter Assay System (Promega, Madison, WI) according to the manufacturer's protocol. Renilla luciferase activity was normalized to firefly luciferase activity and expressed as a percentage of the control. All transfection experiments were performed in triplicate.^11^

### 2.6 Quantitative real‐time RT‐PCR

Total RNA samples were extracted using Trizol (Invitrogen), according to the manufacturer's instructions. Quantitative real‐time reverse transcription PCR (RT‐PCR) analysis was performed using an Applied Biosystems 7500 Real‐Time PCR System (Foster City, Qiagen, CA). The expression level of GAPDH was used as an internal control for messenger RNAs. And the expression level of U6 was used as an internal control for miRNAs.

The primers used in quantitative real‐time PCR analysis are shown in Table 1.

**Table 1:** The primer for gene

Gene	Primer(5′‐3′)	Annealing temperature (°C)	Length (bp)
β‐actin (H)	F:5′ GTGGCCGAGGACTTTGATTG3′	60	73
	R:5′ CCTGTAACAACGCATCTCATATT3′		
hsa_circRNA_101981	F:5′ CGAGGAGCTTATGGTTCTGTCA 3′	60	83
	R:5′ CAGTGCTTTGCGTTTACTTTAACT 3′		
hsa_circRNA_008068	F:5′ TATACCTCTCCCAACAGGTCTC 3′	60	57
	R:5′ TTATCACAAATCTCAGCCAAAT 3′		
hsa_circRNA_406557	F:5′ GAATCCATGTTTCTTCCAAGAA 3′	60	76
	R:5′ TTTGATAAGGCTTCCATACCAT 3′		
hsa_circRNA_091420	F:5′ GGCTGGATTCAGTAATAAATATGT 3′	60	94
	R:5′ AGTCAAGAACACACCACGATG 3′		
hsa_circRNA_101996	F:5′TTACTAAAGGCAAACGGTGAA 3′	60	195
	R:5′GAGTGGGAGTGTTGGAAGAAG3′		

### 2.7 Statistical analysis

All results were the averages of at least 3 independent experiments from separately treated and transfected cultures. Data were expressed as the mean ± SD. Statistical comparisons were performed using 1‐way analysis of variance. *P* < .05 was considered to indicate a statistically signiﬁcant difference.

### 2.8 Analysis of circRNA expression profile

Quantile normalization of raw data and subsequent data processing were performed using the R software limma package. After quantile normalization of the raw data, low‐intensity filtering was performed, and the circRNAs that at least 1 out of 2 samples with flags in “P” or “M” (“All Targets Value”) were retained for further analyses.

Analysis of circRNA differential expression profile When comparing two groups of profile differences (such as disease versus control), the “fold change” (ie the ratio of the group averages) between the groups for each circRNA was computed. The statistical significance of the difference may be conveniently estimated by the *t* test. circRNAs having fold changes ≥2.0 were considered as significantly differentially expressed. One could filter the analysis outputs and rank the differentially expressed circRNAs according to fold change, *P* value, etc, using Microsoft Excel’s Data/Sort & Filter functionalities. The circRNA/miRNA interaction was predicted with Arraystar’s home‐made miRNA target prediction software based on TargetScan^12^ and miRanda,^13^ and the differentially expressed circRNAs within all the comparisons were annotated in detail with the circRNA/miRNA interaction information.

## 3 RESULTS

### 3.1 The successful construction of LNCaP cell lines overexpressing miR‐145

**Figure 1:**
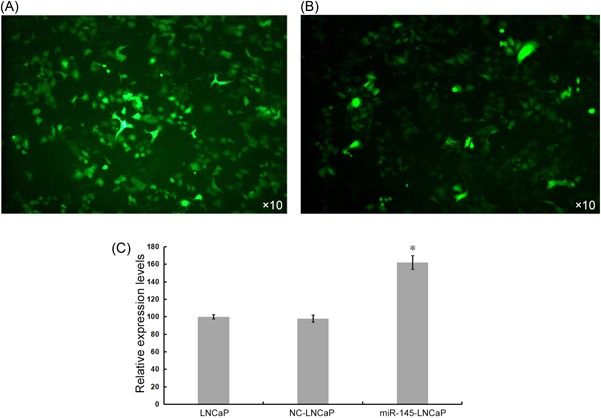
Establishment of overexpression of miR‐145 LNCaP cell line. (A) LNCaP cell line stably transfected with miR‐145 (LNCaP‐miR‐145). (B) LNCaP cell lines stably transfected empty virus (LNCaP‐NC). (C) Relative expression of miR‐145 in LNCaP cells. *P < .01 compared with LNCaP and LNCaP‐NC cell group

The full‐length miR‐145 was cloned into the pLVX‐mCMV‐ZsGreen‐puro vector, and 293T cells were cotransfected with the envelope plasmid pHelper 1.0 and the packaging plasmid pHelper 2.0. Then, the virus titer was measured at 1.25 × 10^8^ TU/mL using dilution method. At MOI = 50, the rlv‐miR‐145 virus was added to the LNCaP cells. Fluorescence microscopy was used to confirm the successfully constructed LNCap cells overexpressing miR‐145 (Figure 1A,B). The RT‐PCR was used to detect the relative expression level of miR‐145. The results showed that the relative expression of miR‐145 was higher than the negative control group. The above results indicated the successful construction of LNCaP cell lines overexpressing miR‐145 (Figure 1C).

### 3.2 Differentially expressed circRNAs detected based on microarray analysis

**Figure 2:**
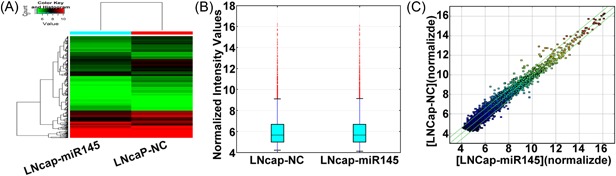
Differential expression of circRNAs in LNCaP cells. (A) Hierarchical clustering analysis of circRNAs that were differentially expressed between LNCaP‐miR‐145 cells and LNCaP‐NC cells; each group contains 3 individuals (greater than 2‐fold difference in expression; *P* < .05). Blue‐white indicates lower expression, and red indicates high expression. (B) The boxplot is a convenient way to quickly visualize the distribution of a data set. It is commonly used for comparing the distributions of the intensities from all samples. Here, a boxplot view is used to look at and compare the distributions of expression values for the samples in an experiment after normalization. (C) The scatter plot is a visualization method used for evaluating the changing expression profiles of circRNAs between LNCaP‐miR‐145 cells and LNCaP‐NC cells. The values corresponding to the *X*‐ and *Y*‐axes in the scatter plot are the normalized signal values of the samples (log2 scaled). The green lines represent fold changes. The circRNAs above the top green line and below the bottom green line represent more than 2.0‐fold changes between LNCaP‐miR‐145 cells and LNCaP‐NC cells. circRNA, circular RNA

Total RNAs from the LNCaP‐miR‐145 and LNCaP‐NC cells were extracted. Arraystar human circRNA Array (815 K V2, Arraystar) was adopted for the detection of circRNA expression, and total of 10 743 circRNAs were detected. Among the detected circRNAs, we found that 267 and 149 circRNAs were significantly up‐ and downregulated in LNCaP‐miR‐145 cells, respectively (Figure 2). By using the bioinformatics software, we were able to identify the chromosome loci and the miRNA targets for 5 of the circRNAs, the expression levels of which were significant different on miR‐145 overexpression. Our result indicated that most of the circRNAs corresponded to the protein‐coding exons. Moreover, hsa_circRNA_101981, hsa_circRNA_101996, and hsa_circRNA_09142 were the 3 circRNAs that interacted with hsa‐miR‐145‐5p; hsa_circRNA_008068 and hsa_circRNA_406557 were the 2 circRNAs that interacted with hsa‐miR‐145‐3p (Table 2).

**Table 2:** Differential expression circRNA

circRNA	Chrom	circRNA_type	Gene Symbol	Bingding miRNA	Expression level
hsa_circRNA_101981	chr17	Exonic	MAP2K4	hsa‐miR‐145‐5p	Up
hsa_circRNA_008068	chr13	Exonic	KATNAL1	hsa‐miR‐145‐3p	Up
hsa_circRNA_406557	chr4	Exonic	RAPGEF2	hsa‐miR‐145‐3p	Up
hsa_circRNA_101996	chr17	Exonic	SPECC1	hsa‐miR‐145‐5p	Down
hsa_circRNA_091420	chrX	Exonic	RPL39	hsa‐miR‐145‐5p	Down

circRNA, circular RNA.

### 3.3 Validation of candidate circRNAs using quantitative RT‐PCR

**Figure 3:**
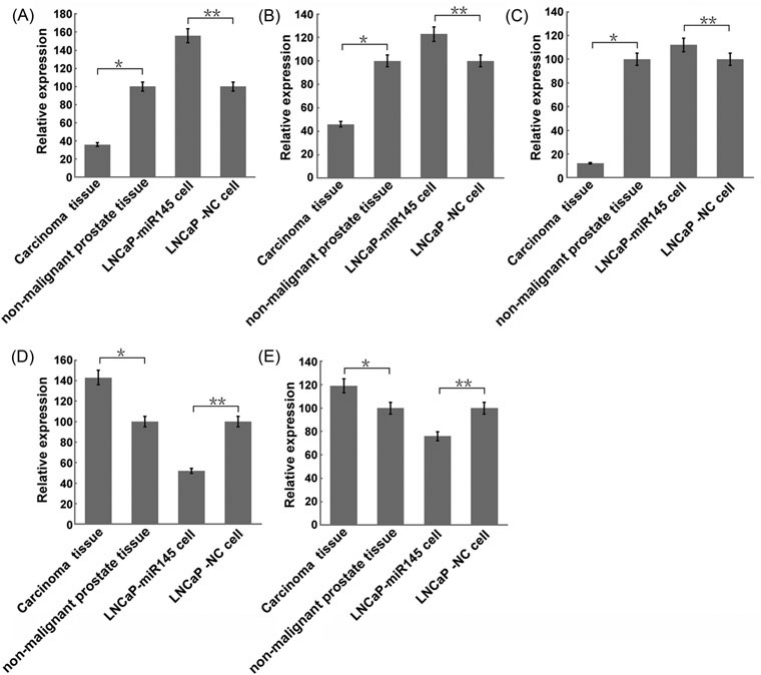
Validation of candidate circRNAs using qRT‐PCR. (A) Expression of hsa_cicRNA_101981 in the 10 paired human PCa tissues, nonmalignant tissues, LNCaP‐miR‐145 cells, and LNCaP‐NC cells by real‐time PCR. Each bar represents the mean of 3 independent experiments. *P < .05, **P < .05. (B) Expression of hsa_cicRNA_008068 in the 10 paired human PCa tissues, nonmalignant tissues, LNCaP‐miR‐145 cells, and LNCaP‐NC cells by real‐time PCR. Each bar represents the mean of 3 independent experiments. *P < .05, **P < .01. (C) Expression of hsa_cicRNA_406557 in the 10 paired human PCa tissues, nonmalignant tissues, LNCaP‐miR‐145 cells, and LNCaP‐NC cells by real‐time PCR. Each bar represents the mean of 3 independent experiments. *P < .01, **P < .01. (D) Expression of hsa_cicRNA_101996 in the 10 paired human PCa tissues, nonmalignant tissues, LNCaP‐miR‐145 cells, and LNCaP‐NC cells by real‐time PCR. Each bar represents the mean of 3 independent experiments. *P < .05, **P < .05 (E) Expression of hsa_cicRNA_091420 in the 10 paired human PCa tissues, nonmalignant tissues, LNCaP‐miR‐145 cells, and LNCaP‐NC cells by real‐time PCR. Each bar represents the mean of 3 independent experiments. *P < .05, **P < .05. circRNA, circular RNA; PCa, prostate cancer; qRT‐PCR, quantitative reverse transcription polymerase chain reaction

Total RNAs from the Pca tissue, nonmalignant tissue, LNCaP‐miR‐145, and LNCaP‐NC cells were extracted, and were further subjected for analysis by quantitative RT‐PCR. The results showed that the expression levels of hsa_circRNA_101981, hsa_circRNA_00806, and hsa_circRNA_406557 were upregulated in the LNCaP‐miR‐145 cells, but downregulated in the Pca tissue. In contrast, the expression levels of hsa_circRNA_101996 and hsa_circRNA_091420 were downregulated in the LNCaP‐miR‐145 cells, but upregulated in the Pca tissue (Figure 3).

### 3.4 Confirmation of the circRNA‐miRNA interaction by the dual‐luciferase reporter assay

**Figure 4:**
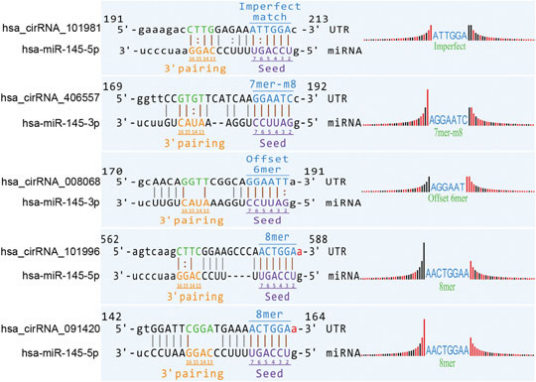
Binding sites of circRNAs and miRNAs. circRNA, circular RNA; miRNA, microRNA

**Figure 5:**
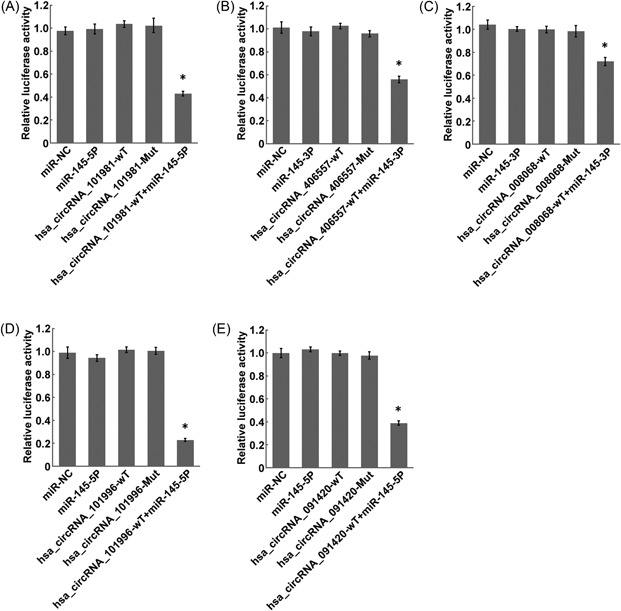
Construction of double fluorescent reporter gene vector to verify the combination of circRNAs and miRNAs. (A) Effects of inhibitor of miR‐145‐5p(miR‐NC), miR‐145‐5P mimics (miR‐145‐5p), hsa_circRNA_101981 wild‐type (hsa_circRNA_101981‐WT), hsa_circRNA_101981 mutation type (hsa_circRNA_101981‐Mut) on the luciferase activity of miR‐145‐5p in LNCap cells by the luciferase reporter assay. Each bar represents the mean of 3 independent experiments. *P < .05. (B) Effects of inhibitor of miR‐145‐3p(miR‐NC), miR‐145‐3P mimics (miR‐145‐3p), hsa_circRNA_406657 wild‐type (hsa_circRNA_406657‐WT), hsa_circRNA_406657 mutation type (hsa_circRNA_406657‐Mut) on the luciferase activity of miR‐145‐5p in LNCap cells by luciferase reporter assay. Each bar represents the mean of 3 independent experiments. *P < .05. (C) Effects of inhibitor of miR‐145‐3p(miR‐NC), miR‐145‐3P mimics(miR‐145‐3p), hsa_circRNA_008068 wild‐type (hsa_circRNA_008068‐WT), hsa_circRNA_008068 mutation type (hsa_circRNA_008068‐Mut) on the luciferase activity of miR‐145‐3p in LNCap cells by luciferase reporter assay. Each bar represents the mean of 3 independent experiments. *P < .05. (D) Effects of inhibitor of miR‐145‐5p(miR‐NC), miR‐145‐5P mimics (miR‐145‐5p), hsa_circRNA_101996 wild‐type (hsa_circRNA_101996‐WT), hsa_circRNA_101996 mutation type (hsa_circRNA_101996‐Mut) on the luciferase activity of miR‐145‐5p in LNCap cells by the luciferase reporter assay. Each bar represents the mean of 3 independent experiments. *P < .01. (E) Effects of inhibitor of miR‐145‐5p(miR‐NC), miR‐145‐5P mimics (miR‐145‐5p), hsa_circRNA_091420 wild‐type (hsa_circRNA_091420‐WT), hsa_circRNA_091420 mutation type (hsa_circRNA_091420‐Mut) on the luciferase activity of miR‐145‐5p in LNCap cells by luciferase reporter assay. Each bar represents the mean of 3 independent experiments. *P < .05. circRNA, circular RNA

To further confirm the interaction of circRNA and the target miRNA, the bioinformatics software TargetScan and miRanda were used (Figure 4). On the identification of the interaction, the expression vectors containing wild‐type or mutant circRNA were constructed and then cotransfected with miRNA mimics into the LNCaP cells. The fluorescence activities were then measured 48 hours after transfection. The results showed that the fluorescence activities were significantly decreased in cells cotransfected with: (1) miR‐145‐5P mimics and expression vectors containing wild‐type hsa_circRNA_101981, hsa_circRNA_101996, or hsa_circRNA_091420; (2) miR‐145‐3P mimics and expression vectors containing wild‐type hsa_circRNA_008068 or hsa_circRNA_406557. It suggested that the miR‐145 mimics might participate in the regulation of the identified circRNAs (Figure 5). On the contrary, the fluorescence activities showed no obvious change in cells cotransfected with the miR‐145 mimics and the mutant circRNAs, suggesting that miR‐145 could not regulate the expression of the mutated circRNAs.

## 4 DISCUSSION

cirRNA, a type of newly discovered endogenous long noncoding RNA, has been shown to play an important role in gene regulation, and thus received a lot of research attention.^14^ Unlike the linear RNA that contains the 5′ cap and a 3′ poly(A) tail, circRNA is generated via back‐splicing of messenger RNA and forms a covalently closed continuous loop by joining the 3′ and 5′ end, and thus more stable in cells.^15^ It is largely expressed in eukaryotic transcriptome, which is conserved among species with stable expression and tissue/time specificity. A recent study has shown that circRNA could function as a miRNA sponge that interacted with RNA or protein and participate in the regulation of gene transcription.^16^

In this study, we investigated the circRNA expression alterations of LNCap cells. This is the very first study to explore the expression profile of circRNAs in Pca cells with overexpression of miR‐145. A large number of circRNAs were aberrantly expressed in LNCap‐miR‐145 cells, suggesting that these circRNAs might have roles in Pca, and that some circRNAs validated in our study might be used as a potential biomarker or therapeutic target for the treatment of Pca.

miR‐145 is considered as a tumor suppressor in different types of cancer. miR‐145 was located on chromosome5q32chr5: 149430646‐149430733 [+]. Its stem ring structure was cut into mature sequences by the action of DICER enzyme: one is miR‐145‐5p (MIMAT0000437;GUCCAGUUUUCCCAGGAAUCCCU), and the other is miR‐145‐3p (MIMAT0004601; GGAUUCCUGGAAAUACUGUUCU.

miR‐145‐5p could suppress cell proliferation, invasion, and migration and induce apoptosis of CHL‐1 and VMM917 melanoma cells by inhibiting mitogen‐activated protein kinas (MAPK) and phosphatid‐ylinositol 3 kinase/protein kinae B (P13k/AKT) pathways.^16^ It might also function as a cardiac‐protective molecule in myocardial ischemic injury by ameliorating inflammation and apoptosis via negative regulation of CD40.^17^ miR‐145 was also shown to mediate epithelial‐mesenchyma transition (EMT) by targeting Snail and it might be a novel EMT regulating transcription factor that involved in the progression of osteogenic system (OS).^18^ miR‐145 negatively regulated the invasion of cell lines through targeting N‐cadherin by directly binding to its 3′‐untranslated region. Silencing of N‐cadherin inhibited invasion and migration of LAC cell lines, which was also observed when miR‐145 was overexpressed.^19^ miR‐145 was able to induce computed tomography differentiation through enzymolyzing transcript factors (TFs) and might be a therapeutic target for cervical carcinoma. It could also promote hematopoietic stem cell activation and liver fibrosis by targeting KLF4.^20^ Our previous result has shown that miR‐145 not only could inhibit the proliferation and invasion of Pca LNCaP cells, but also involved in the regulation of lncRNAPCGEM1.^3^ However, whether the interaction of miR‐145 and cirRNA are involved in the pathogenesis of Pca remains to be further elucidated.

An increasing number of studies have shown that miRNA could suppress the degradation of the corresponding messenger RNA by competitively binding to exonic circRNA at its binding site.^8^ In bladder cancer, circRNA MYLK and long noncoding RNA H19 competitively binded to miR‐29a‐3P as endogenous RNAs, which further upregulated the gene expression of DNMT3, VEGFA, and ITGB1(Mengge^21^); circHIPK3 inhibited heparanase expression through competitive binding to miR‐558.^22^ circ‐0005075 was shown to be highly expressed in liver cancer tissues.^23^ Our results suggested that miR‐145‐5P might regulate the expression of hsa_circRNA_101981, hsa_circRNA_101996, and hsa_circRNA_09142, while miR‐145‐3P might regulate the expression of hsa_circRNA_008068 and hsa_circRNA_406557. Overexpression of miR‐145 could promote the expression of hsa_circRNA_101981, hsa_circRNA_008068, and hsa_circRNA_406557, but suppress the expression of hsa_circRNA_101996 and hsa_circRNA_091420 in LNCaP cells.

In conclusion, our study adds important knowledge to the field because there is no similar study in Pca LNCap cells up to date. The identification of the novel alteration in circRNAs expression is the first crucial step toward the better understanding of the role of circRNAs in acute respiratory distress syndrome. Then, deciphering the precise molecular mechanisms of circRNA function will be critical for understanding PCa pathogenesis and developing new potential therapeutic targets. One may predict that more work is needed to explore the role of circRNAs in PCa in the near future.

## CONFLICTS OF INTEREST

The authors declare that they have no conflict of interests.

## Supporting information

Supporting information
