# Supplementary material for: MiR‐145 affected the circular RNA expression in prostate cancer LNCaP cells
Source: J Cell Biochem. 2018 Aug 22;119(11):9168–77. doi: 10.1002/jcb.27181 (PMC6704360; doi:10.1002/jcb.27181)
Supplement: Supplementary file 1 — Supporting information [file JCB-119-9168-s001.docx]

Annexed table 1. Different expression of circRNA in LNCaP cell after overexpression of miR-145

| up regulation |  | down regulation |
| --- | --- | --- |
| hsa_circRNA_006349 |  | hsa_circRNA_407279 |
| hsa_circRNA_003596 |  | hsa_circRNA_100751 |
| hsa_circRNA_400185 |  | hsa_circRNA_101408 |
| hsa_circRNA_089761 |  | hsa_circRNA_100445 |
| hsa_circRNA_404497 |  | hsa_circRNA_038032 |
| hsa_circRNA_101981 |  | hsa_circRNA_089978 |
| hsa_circRNA_003147 |  | hsa_circRNA_008584 |
| hsa_circRNA_061481 |  | hsa_circRNA_406325 |
| hsa_circRNA_008068 |  | hsa_circRNA_010096 |
| hsa_circRNA_403389 |  | hsa_circRNA_400031 |
| hsa_circRNA_401955 |  | hsa_circRNA_406213 |
| hsa_circRNA_084903 |  | hsa_circRNA_046753 |
| hsa_circRNA_104528 |  | hsa_circRNA_405602 |
| hsa_circRNA_103046 |  | hsa_circRNA_079352 |
| hsa_circRNA_405521 |  | hsa_circRNA_000410 |
| hsa_circRNA_016771 |  | hsa_circRNA_011491 |
| hsa_circRNA_082778 |  | hsa_circRNA_001955 |
| hsa_circRNA_103213 |  | hsa_circRNA_015063 |
| hsa_circRNA_102319 |  | hsa_circRNA_001886 |
| hsa_circRNA_100003 |  | hsa_circRNA_101760 |
| hsa_circRNA_104670 |  | hsa_circRNA_405868 |
| hsa_circRNA_075671 |  | hsa_circRNA_006766 |
| hsa_circRNA_104543 |  | hsa_circRNA_101296 |
| hsa_circRNA_050649 |  | hsa_circRNA_405563 |
| hsa_circRNA_048764 |  | hsa_circRNA_405002 |
| hsa_circRNA_404462 |  | hsa_circRNA_014225 |
| hsa_circRNA_404768 |  | hsa_circRNA_036052 |
| hsa_circRNA_103283 |  | hsa_circRNA_000997 |
| hsa_circRNA_104532 |  | hsa_circRNA_101838 |
| hsa_circRNA_101300 |  | hsa_circRNA_004936 |
| hsa_circRNA_101795 |  | hsa_circRNA_102361 |
| hsa_circRNA_406652 |  | hsa_circRNA_025501 |
| hsa_circRNA_001530 |  | hsa_circRNA_010906 |
| hsa_circRNA_028826 |  | hsa_circRNA_102322 |
| hsa_circRNA_004117 |  | hsa_circRNA_062898 |
| hsa_circRNA_405434 |  | hsa_circRNA_072697 |
| hsa_circRNA_405709 |  | hsa_circRNA_076905 |
| hsa_circRNA_009618 |  | hsa_circRNA_067926 |
| hsa_circRNA_006675 |  | hsa_circRNA_100882 |
| hsa_circRNA_104659 |  | hsa_circRNA_407287 |
| hsa_circRNA_402690 |  | hsa_circRNA_034435 |
| hsa_circRNA_002545 |  | hsa_circRNA_104263 |
| hsa_circRNA_036567 |  | hsa_circRNA_001449 |
| hsa_circRNA_405474 |  | hsa_circRNA_406237 |
| hsa_circRNA_400029 |  | hsa_circRNA_404013 |
| hsa_circRNA_100278 |  | hsa_circRNA_089039 |
| hsa_circRNA_405551 |  | hsa_circRNA_049241 |
| hsa_circRNA_101721 |  | hsa_circRNA_101996 |
| hsa_circRNA_102527 |  | hsa_circRNA_104802 |
| hsa_circRNA_102531 |  | hsa_circRNA_102563 |
| hsa_circRNA_406828 |  | hsa_circRNA_008828 |
| hsa_circRNA_104615 |  | hsa_circRNA_405564 |
| hsa_circRNA_100240 |  | hsa_circRNA_101773 |
| hsa_circRNA_052621 |  | hsa_circRNA_104004 |
| hsa_circRNA_103271 |  | hsa_circRNA_103462 |
| hsa_circRNA_406038 |  | hsa_circRNA_103532 |
| hsa_circRNA_100667 |  | hsa_circRNA_043650 |
| hsa_circRNA_018834 |  | hsa_circRNA_405028 |
| hsa_circRNA_008817 |  | hsa_circRNA_407326 |
| hsa_circRNA_404514 |  | hsa_circRNA_023523 |
| hsa_circRNA_001100 |  | hsa_circRNA_027023 |
| hsa_circRNA_103670 |  | hsa_circRNA_052282 |
| hsa_circRNA_037798 |  | hsa_circRNA_015962 |
| hsa_circRNA_101553 |  | hsa_circRNA_051908 |
| hsa_circRNA_400223 |  | hsa_circRNA_402028 |
| hsa_circRNA_100098 |  | hsa_circRNA_001418 |
| hsa_circRNA_404454 |  | hsa_circRNA_103777 |
| hsa_circRNA_059060 |  | hsa_circRNA_092570 |
| hsa_circRNA_102517 |  | hsa_circRNA_092558 |
| hsa_circRNA_104336 |  | hsa_circRNA_100662 |
| hsa_circRNA_406752 |  | hsa_circRNA_103842 |
| hsa_circRNA_038737 |  | hsa_circRNA_000827 |
| hsa_circRNA_102251 |  | hsa_circRNA_101555 |
| hsa_circRNA_002144 |  | hsa_circRNA_400915 |
| hsa_circRNA_103756 |  | hsa_circRNA_101231 |
| hsa_circRNA_103361 |  | hsa_circRNA_407330 |
| hsa_circRNA_101525 |  | hsa_circRNA_401506 |
| hsa_circRNA_000625 |  | hsa_circRNA_103839 |
| hsa_circRNA_102762 |  | hsa_circRNA_000780 |
| hsa_circRNA_003063 |  | hsa_circRNA_092556 |
| hsa_circRNA_007854 |  | hsa_circRNA_400692 |
| hsa_circRNA_100762 |  | hsa_circRNA_049729 |
| hsa_circRNA_100433 |  | hsa_circRNA_405111 |
| hsa_circRNA_001198 |  | hsa_circRNA_003223 |
| hsa_circRNA_103409 |  | hsa_circRNA_101123 |
| hsa_circRNA_001618 |  | hsa_circRNA_036391 |
| hsa_circRNA_103272 |  | hsa_circRNA_002009 |
| hsa_circRNA_100715 |  | hsa_circRNA_402915 |
| hsa_circRNA_020256 |  | hsa_circRNA_104017 |
| hsa_circRNA_100527 |  | hsa_circRNA_103551 |
| hsa_circRNA_104707 |  | hsa_circRNA_100822 |
| hsa_circRNA_101573 |  | hsa_circRNA_101966 |
| hsa_circRNA_004665 |  | hsa_circRNA_017516 |
| hsa_circRNA_104372 |  | hsa_circRNA_404473 |
| hsa_circRNA_001126 |  | hsa_circRNA_104463 |
| hsa_circRNA_100120 |  | hsa_circRNA_101919 |
| hsa_circRNA_009052 |  | hsa_circRNA_104626 |
| hsa_circRNA_049055 |  | hsa_circRNA_049537 |
| hsa_circRNA_100591 |  | hsa_circRNA_077032 |
| hsa_circRNA_403587 |  | hsa_circRNA_103243 |
| hsa_circRNA_100245 |  | hsa_circRNA_004868 |
| hsa_circRNA_006473 |  | hsa_circRNA_032602 |
| hsa_circRNA_405304 |  | hsa_circRNA_001750 |
| hsa_circRNA_009548 |  | hsa_circRNA_065673 |
| hsa_circRNA_032377 |  | hsa_circRNA_404802 |
| hsa_circRNA_038901 |  | hsa_circRNA_091420 |
| hsa_circRNA_403676 |  | hsa_circRNA_103168 |
| hsa_circRNA_104114 |  | hsa_circRNA_101220 |
| hsa_circRNA_103539 |  | hsa_circRNA_101888 |
| hsa_circRNA_101777 |  | hsa_circRNA_402169 |
| hsa_circRNA_103716 |  | hsa_circRNA_100313 |
| hsa_circRNA_060067 |  | hsa_circRNA_102890 |
| hsa_circRNA_102358 |  | hsa_circRNA_004885 |
| hsa_circRNA_004546 |  | hsa_circRNA_001226 |
| hsa_circRNA_102888 |  | hsa_circRNA_403802 |
| hsa_circRNA_008561 |  | hsa_circRNA_102351 |
| hsa_circRNA_032891 |  | hsa_circRNA_035775 |
| hsa_circRNA_063089 |  | hsa_circRNA_406111 |
| hsa_circRNA_006062 |  | hsa_circRNA_101283 |
| hsa_circRNA_400987 |  | hsa_circRNA_103246 |
| hsa_circRNA_100711 |  | hsa_circRNA_103546 |
| hsa_circRNA_405661 |  | hsa_circRNA_102306 |
| hsa_circRNA_003782 |  | hsa_circRNA_100335 |
| hsa_circRNA_405192 |  | hsa_circRNA_103604 |
| hsa_circRNA_101839 |  | hsa_circRNA_102657 |
| hsa_circRNA_102030 |  | hsa_circRNA_101303 |
| hsa_circRNA_003819 |  | hsa_circRNA_406001 |
| hsa_circRNA_103682 |  | hsa_circRNA_405849 |
| hsa_circRNA_103739 |  | hsa_circRNA_087386 |
| hsa_circRNA_104137 |  | hsa_circRNA_040629 |
| hsa_circRNA_004825 |  | hsa_circRNA_015382 |
| hsa_circRNA_404658 |  | hsa_circRNA_401622 |
| hsa_circRNA_102029 |  | hsa_circRNA_001923 |
| hsa_circRNA_100583 |  | hsa_circRNA_001914 |
| hsa_circRNA_101492 |  | hsa_circRNA_404825 |
| hsa_circRNA_034805 |  | hsa_circRNA_103488 |
| hsa_circRNA_101128 |  | hsa_circRNA_103493 |
| hsa_circRNA_037558 |  | hsa_circRNA_103393 |
| hsa_circRNA_100032 |  | hsa_circRNA_104838 |
| hsa_circRNA_000076 |  | hsa_circRNA_005800 |
| hsa_circRNA_400071 |  | hsa_circRNA_002873 |
| hsa_circRNA_000023 |  | hsa_circRNA_404530 |
| hsa_circRNA_000274 |  | hsa_circRNA_100155 |
| hsa_circRNA_104248 |  | hsa_circRNA_104682 |
| hsa_circRNA_101405 |  | hsa_circRNA_039933 |
| hsa_circRNA_103530 |  | hsa_circRNA_104169 |
| hsa_circRNA_006383 |  | hsa_circRNA_076734 |
| hsa_circRNA_079265 |  | hsa_circRNA_072837 |
| hsa_circRNA_104859 |  | hsa_circRNA_104262 |
| hsa_circRNA_102135 |  | hsa_circRNA_007698 |
| hsa_circRNA_101691 |  |  |
| hsa_circRNA_005019 |  |  |
| hsa_circRNA_017215 |  |  |
| hsa_circRNA_102923 |  |  |
| hsa_circRNA_041939 |  |  |
| hsa_circRNA_000525 |  |  |
| hsa_circRNA_100244 |  |  |
| hsa_circRNA_104327 |  |  |
| hsa_circRNA_100235 |  |  |
| hsa_circRNA_038288 |  |  |
| hsa_circRNA_407176 |  |  |
| hsa_circRNA_072303 |  |  |
| hsa_circRNA_063981 |  |  |
| hsa_circRNA_100543 |  |  |
| hsa_circRNA_082672 |  |  |
| hsa_circRNA_103780 |  |  |
| hsa_circRNA_000494 |  |  |
| hsa_circRNA_103035 |  |  |
| hsa_circRNA_401701 |  |  |
| hsa_circRNA_001161 |  |  |
| hsa_circRNA_105050 |  |  |
| hsa_circRNA_101202 |  |  |
| hsa_circRNA_400244 |  |  |
| hsa_circRNA_404446 |  |  |
| hsa_circRNA_068465 |  |  |
| hsa_circRNA_047274 |  |  |
| hsa_circRNA_086475 |  |  |
| hsa_circRNA_104851 |  |  |
| hsa_circRNA_100533 |  |  |
| hsa_circRNA_003181 |  |  |
| hsa_circRNA_004428 |  |  |
| hsa_circRNA_400105 |  |  |
| hsa_circRNA_402089 |  |  |
| hsa_circRNA_001589 |  |  |
| hsa_circRNA_073248 |  |  |
| hsa_circRNA_005161 |  |  |
| hsa_circRNA_002889 |  |  |
| hsa_circRNA_104940 |  |  |
| hsa_circRNA_103376 |  |  |
| hsa_circRNA_026686 |  |  |
| hsa_circRNA_103619 |  |  |
| hsa_circRNA_101878 |  |  |
| hsa_circRNA_100894 |  |  |
| hsa_circRNA_102336 |  |  |
| hsa_circRNA_101992 |  |  |
| hsa_circRNA_105035 |  |  |
| hsa_circRNA_404576 |  |  |
| hsa_circRNA_015524 |  |  |
| hsa_circRNA_406007 |  |  |
| hsa_circRNA_012451 |  |  |
| hsa_circRNA_404096 |  |  |
| hsa_circRNA_104547 |  |  |
| hsa_circRNA_048766 |  |  |
| hsa_circRNA_103155 |  |  |
| hsa_circRNA_042368 |  |  |
| hsa_circRNA_064743 |  |  |
| hsa_circRNA_103138 |  |  |
| hsa_circRNA_101749 |  |  |
| hsa_circRNA_101233 |  |  |
| hsa_circRNA_104046 |  |  |
| hsa_circRNA_018499 |  |  |
| hsa_circRNA_102480 |  |  |
| hsa_circRNA_036599 |  |  |
| hsa_circRNA_006776 |  |  |
| hsa_circRNA_402737 |  |  |
| hsa_circRNA_051695 |  |  |
| hsa_circRNA_048977 |  |  |
| hsa_circRNA_402191 |  |  |
| hsa_circRNA_005522 |  |  |
| hsa_circRNA_092417 |  |  |
| hsa_circRNA_100143 |  |  |
| hsa_circRNA_103540 |  |  |
| hsa_circRNA_013533 |  |  |
| hsa_circRNA_102245 |  |  |
| hsa_circRNA_400064 |  |  |
| hsa_circRNA_406166 |  |  |
| hsa_circRNA_102908 |  |  |
| hsa_circRNA_102338 |  |  |
| hsa_circRNA_102876 |  |  |
| hsa_circRNA_100853 |  |  |
| hsa_circRNA_102981 |  |  |
| hsa_circRNA_004646 |  |  |
| hsa_circRNA_400275 |  |  |
| hsa_circRNA_104053 |  |  |
| hsa_circRNA_103595 |  |  |
| hsa_circRNA_032969 |  |  |
| hsa_circRNA_103437 |  |  |
| hsa_circRNA_406620 |  |  |
| hsa_circRNA_102033 |  |  |
| hsa_circRNA_103421 |  |  |
| hsa_circRNA_402867 |  |  |
| hsa_circRNA_100375 |  |  |
| hsa_circRNA_001059 |  |  |
| hsa_circRNA_104814 |  |  |
| hsa_circRNA_103521 |  |  |
| hsa_circRNA_100544 |  |  |
| hsa_circRNA_051239 |  |  |
| hsa_circRNA_103225 |  |  |
| hsa_circRNA_069837 |  |  |
| hsa_circRNA_027691 |  |  |
| hsa_circRNA_104671 |  |  |
| hsa_circRNA_104815 |  |  |
| hsa_circRNA_406875 |  |  |
| hsa_circRNA_406192 |  |  |
| hsa_circRNA_068697 |  |  |
| hsa_circRNA_007472 |  |  |
| hsa_circRNA_102787 |  |  |
| hsa_circRNA_103000 |  |  |
| hsa_circRNA_065768 |  |  |
| hsa_circRNA_400629 |  |  |
| hsa_circRNA_406557 |  |  |
| hsa_circRNA_081968 |  |  |
| hsa_circRNA_000042 |  |  |
| hsa_circRNA_000328 |  |  |
| hsa_circRNA_403751 |  |  |
| hsa_circRNA_404289 |  |  |
| hsa_circRNA_102518 |  |  |
| hsa_circRNA_020704 |  |  |
